# Identification of a hippocampal lncRNA-regulating network in a natural aging rat model

**DOI:** 10.1186/s12868-022-00743-7

**Published:** 2022-09-28

**Authors:** Li Qi, Xiao Li, Shi-min Liu, Dan-li Jiao, Dan Hu, Xin-yao Ju, Shu-yu Zhao, Shu-han Si, Li Hu, Guo-na Li, Bing-zhe Ma, Shuang Zhou, Chen Zhao

**Affiliations:** 1 Institute of Interdisciplinary Integrative Medicine Research, Shanghai University of Traditional Chinese Medicine, Shanghai, 201203 China; 2 College of Acumox and Tuina, Shanghai University of Traditional Chinese Medicine, Shanghai, 201203 China; 3 Yueyang Hospital of Integrated Chinese and Western Medicine, Shanghai University of Traditional Chinese Medicine, Shanghai, 200437 China

**Keywords:** Aging, mRNAs, lncRNAs, Hippocampus

## Abstract

**Background:**

Dysregulation of long noncoding RNA (lncRNA) expression is related to aging and age-associated neurodegenerative diseases, and the lncRNA expression profile in the aging hippocampus is not well characterized. In the present investigation, the changed mRNAs and lncRNAs were confirmed via deep RNA sequencing. GO and KEGG pathway analyses were conducted to investigate the principal roles of the clearly dysregulated mRNAs and lncRNAs. Subsequently, through the prediction of miRNAs via which mRNAs and lncRNAs bind together, a competitive endogenous RNA network was constructed.

**Results:**

A total of 447 lncRNAs and 182 mRNAs were upregulated, and 385 lncRNAs and 144 mRNAs were downregulated. Real-time reverse transcription-polymerase chain reaction validated the reliability of mRNA and lncRNA sequencing. KEGG pathway and GO analyses revealed that differentially expressed (DE) mRNAs were associated with cell adhesion molecules (CAMs), the p53 signaling pathway (SP), phagosomes, PPAR SP and ECM—receptor interactions. KEGG pathway and GO analyses showed that the target genes of the DE lncRNAs were related to cellular senescence, the p53 signaling pathway, leukocyte transendothelial migration and tyrosine metabolism. Coexpression analyses showed that 561 DE lncRNAs were associated with DE mRNAs. A total of 58 lncRNA–miRNA–mRNA target pairs were confirmed in this lncRNA‒miRNA‒mRNA network, comprising 10 mRNAs, 13 miRNAs and 38 lncRNAs.

**Conclusions:**

We found specific lncRNAs and mRNAs in the hippocampus of natural aging model rats, as well as abnormal regulatory ceRNA networks. Our outcomes help explain the pathogenesis of brain aging and provide direction for further research.

**Supplementary Information:**

The online version contains supplementary material available at 10.1186/s12868-022-00743-7.

## Introduction

As the global population ages, problems related to aging are sparking immense attention [1, 2]. Brain aging processes are highly complex phenomena. Many basic and clinical investigations have shown that the hippocampus is the main part of the brain involved in aging and dementia [3]. Changes in aging brains comprise changes in the transcription and epigenetics of coding and noncoding genome areas. Among noncoding transcripts, long noncoding RNAs (lncRNAs) have recently emerged as essential regulators of the molecular pathways underlying age-related phenotypes. Previous investigations have also demonstrated that numerous alterations in lncRNA expression occur during aging [4, 5], and it remains unknown whether the lncRNA-modulating network in the hippocampus is altered and how it changes.

lncRNAs are a type of RNAs that are over 200 nucleotides in length, and they lack a complete open reading frame, featuring no or little protein-coding capability. lncRNAs modulate gene expression via various mechanisms, such as RNA–DNA interactions, RNA–protein interactions and RNA–RNA base pairing [6]. In the last twenty years, it has been indicated that lncRNAs, featuring specific spatiotemporal expression patterns across different species, are broadly involved in many biological pathways, including posttranscriptional processing, transcription control, chromatin remodeling and epigenetic regulation [7, 8]. Recent investigations have shown that hundreds of lncRNAs undergo significant changes during the aging process in many organisms, including rhesus monkeys [9] and *C. elegans* [10]. Additional investigations have confirmed that lncRNAs are involved in the pathogenesis of various age-related disorders, such as liver cancer [11], colorectal cancer [12], vascular aging [13], and Parkinson’s disease [14], implying that lncRNAs play a role in the growth and aging of various tissues and organs. Previous studies have shown that, at the epigenetic level, lncRNAs in the hippocampus are closely related to various age-related neuropsychiatric diseases [15], suggesting that lncRNAs in the hippocampus may play an important role in brain aging.

The rat model of natural aging is an ideal animal model for aging research. It can accurately and completely reflect the aging state of the body and the characteristics of human aging [16]. Our previous studies showed that, compared with young rats, the expression of P16 protein and senescence-associated β-galactosidase (sa-β-gal) in the hippocampus of aging rats were increased [17]. Other investigations have shown that the learning and memory ability of aging rats was decreased in the natural aging rat model compared with young rats [18–20]. In this study, we investigated the mRNA and lncRNA expression profiles in the hippocampal tissue of aging rats and constructed a lncRNA–miRNA–mRNA competing endogenous RNA (ceRNA) network by adopting RNA sequencing technology to offer a new theoretical foundation for targeted remedies for aging.

## Materials and methods

### Experimental animals

Male Sprague–Dawley (SD) rats (9 months old or 14 months old, n = 3) were provided and fed as prescribed by the Animal Centre of Shanghai University of Traditional Chinese Medicine (TCM), Shanghai, China. Animals were housed in an environmentally controlled feeding room (with free access to water and food, 20 ± 2 °C, 12 h light/dark cycle). The investigation was permitted by the Animal Ethics Committee of the Shanghai University of TCM. This study was carried out in strict accordance with the recommendations in the National Laboratory Animal Management Regulations of China.

### Natural aging rat model

Fourteen-month-old rats were raised to 20 months of age.

### Tissue collection

The rats were killed through cervical vertebral dislocation after anesthetization using pentobarbital sodium, and then the brains were rapidly excised. The gathered specimens were washed with cold normal saline. Then, the hippocampal region was divided, frozen in liquid N_2_ and keptat − 80 °C before use. Three samples per group were then subjected to high-throughput sequencing and RT-PCR.

### RNA extraction and library preparation

Total RNA was extracted with an RNeasy Mini Kit (Cat#74106, Qiagen), and RNA quality was checked by applying an Agilent Bioanalyser 4200 (Agilent Technologies, Santa Clara, California, US). The qualifying extracted hippocampal RNA specimenssatisfied the following conditions: the RNA concentration was at least 100 µg/µL; the RNA quantity was at least 1 μg; the OD260/280 value (an indicator of RNA purity) was between 1.8 and 2.2; and the RNA integrity score was at least 7 (RIN ≥ 7). The synthesized cDNA was end-repaired and then subjected to 3′adenylation. The ends of these 3′adenylated cDNA fragments were connected by utilizing adaptors. PCR Master Mix and PCR Primer Cocktail were used for PCR amplification to enrich cDNA fragments. Then, the PCR product was purified using Ampure XP beads. Sequencing libraries were generated using a VAHTSTM Total RNA-seq Library Prep Kit for Illumina (NR603, Vazyme, Nanjing, China) (VAHTSTM Stranded mRNA-seq Library Prep Kit for Illumina (NR612, Vazyme, Nanjing, China) according to the manufacturer’s instructions.

### RNA sequencing and differentially expressed RNA analyses

Sequencing was performed on an Illumina NovaSeq platform (Illumina, San Diego, CA, USA). The Read Counts of transcripts and lncRNAs were calculated by Stringtie(version:1.3.0). And the expression of mRNAs and lncRNAs were normalized to FPKM. Then, the mRNAs and lncRNAs were used for differential expression screening and expression level calculation, and genes with |log2(Fold-change)|≥ 1 and *P* value < 0.05 were considered differentially expressed (DE) genes. EdgeR (version 4.0.1) was run to screen DE genes. Each sequencing course and analysis was performed by Shanghai Biochip Co., Ltd. (Shanghai, China).

### Validation by quantitative real-time polymerase chain reaction (RT‒PCR)

To verify the validity and accuracy of the RNA sequencing results, we carried out RT-PCR assessment to assess data consistency between RNA sequencing and RT‒PCR. Total RNA from 50 mg of hippocampal tissue was extracted using Trizol Reagent according to the manufacturer’s protocol. First-strand complementary DNA (cDNA) was synthesized from total RNA using a First-strand cDNA Synthesis Kit (TOYOBO ReverTra Ace qPCR RT Kit). The SYBR GREEN Mix (ABI Power SYBR Green PCR Master Mix) reaction system was used for RT‒PCR along with a forward primer, a reverse primer, and cDNA. The reaction process included the following steps: (1) a preincubation step at 95 °C for 10 min; (2) an amplification step involving 40 cycles of 95 °C for 15 s, and (3) different annealing temperatures and 60 °C for 1 min. A melting curve was recorded to verify the absence of primer dimers. Glyceraldehyde 3-phosphate dehydrogenase (GAPDH) was the endogenous control. RNA levels were assayed using the “ΔΔ Ct method” for relative expression [21]. The primers used in RT‒PCR are listed in Table 1.

**Table 1:** PCR primers used in this study

Primer Name	Sequence
Cdkn1a	F GACCTAAGCGTACCGTCCAG
	R CCTGTGTACCCGTTCCCTTC
Ifi27	F GCTGGCACCGTTTTATCCAG
	R GCTAGAGAGGAGGCTGCAAT
NONRATT000231.2	F AGCTGAGAGTAGCCTCCACA
	R CTCTACAGTTAGCCCTGCCG
MSTRG.548.1	F TAGACCTAAACTGTCACAAGGTC
	R GAGGTCGTTCAATAGTGGGCT
Mt-cyb	F AACGCAGCTTAACATTCCGC
	R TGGGTGTTCTACTGGTTGGC
NONRATT020704.2	F CTCTCATGCCACTGACACACC
	R CCAGACAGTGGACTCCTATCCTA
MSTRG.6345.3	F AGGCTCAGGTAACGCGTATT
	R GCTAACCTAGTCCGAAGCCA
GAPDH	F GTTGTCTCCTGCGACTTCA
	R TGGTCCAGGGTTTCTTACTC

The primer synthesis was completed by Shanghai Bioengineering Co., Ltd

### Target prediction

Cis and trans regulation analyses were performed to predict the underlying relationships between the altered lncRNAs and mRNAs, with 10 kb as the cutoff in the cis regulation analysis. lncRNAs may affect gene expression by playing cis and trans regulatory roles. Here, only the differentially expressed lncRNAs and mRNAs were used in the prediction to explore the potential function of lncRNAs. The mRNAs within 10 kb upstream and downstream of lncRNAs were selected as potential cis regulators. The corresponding gene sequence database was used to predict trans target genes. The complementary or similar sequences were selected by BLAST, and then the complementary binding energy between the two sequences was calculated by RNAplex to predict the trans target gene.

### GO function and KEGG pathway annotation analyses

All DE genes were mapped to terms in the Kyoto Encyclopedia of Genes and Genomes (KEGG) database (http://www.genome.jp/kegg/pathway.html) and the Gene Ontology database (http://www.geneontology.org/). GO terms and KEGG pathways with *P* < 0.05 were deemed as significantly enriched GO terms and KEGG terms [22].

### LncRNA-mRNA coexpression analyses

For coexpression analysis of lncRNAs and genes, according to the expression levels of DE lncRNAs and genes, the Pearson correlation analysis test was used to calculate the correlation between the two expression levels [21]. A correlation coefficient (COR) of > 0.9 and a *P* value < 0.01 were considered to indicate a coexpression relationship.

### Construction of the lncRNA‒miRNA‒mRNA ceRNA network

The miRbase database and the miRanda program (v. 3.3a) were used to predict the binding between these miRNA-differentially expressed mRNA/differentially expressed lncRNA sequences using the default parameters of miRanda v. 3.3a (S ≥ 150, ΔG ≤  − 30 kcal/mol and demand strict 5’seed pairing), which predicts the miRNAs bound to lncRNAs or mRNAs and then determined the intersection to identify miRNAs bound to both. S refers to the single residue pair match scores of the matching area, and ΔG refers to the free energy of double chain binding [23]. Then, the miRNAs, lncRNAs and mRNAs were used to construct a ceRNA regulatory network of lncRNAs‒miRNAs‒mRNAs using Cytoscape software [24].

### Statistical analysis

Data were analyzed using SPSS 20.0 statistical software (SPSS Inc., Chicago, IL, USA). P < 0.05 was considered to indicate a statistically significant difference. Fold changes and *P* values were used to determine the statistical significance of RNA sequence data. |log2(Fold-change)|≥ 1 and *P* value < 0.05 were used as thresholds for DE lncRNAs and mRNAs.

## Results

### Expression profile of mRNAs in the hippocampus in aging rats

We employed RNA sequencing to explore the mRNA and lncRNA changes in the hippocampus that are closely related to aging. A total of 32,888 mRNAs and 25,092 lncRNAs were identified and subsequently analyzed in depth.

**Fig. 1:**
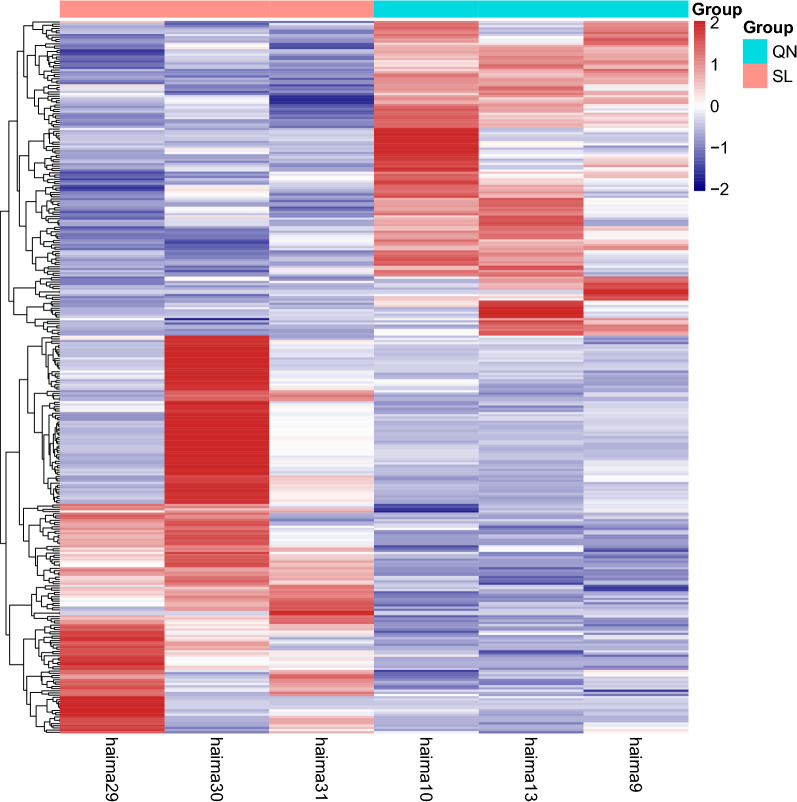
Heatmap of all differentially expressed mRNAs in hippocampal tissues with aging. QN: 9-month control group; SL: aging group (n = 3). Each row represents one mRNA, and each column represents one hippocampal sample. The relative mRNA level is shown by the color scale. Red and blue colors represent high and low relative expression levels, respectively. The fold changes were normalized and scaled from − 2.0 to 2.0 by Z score

**Fig. 2:**
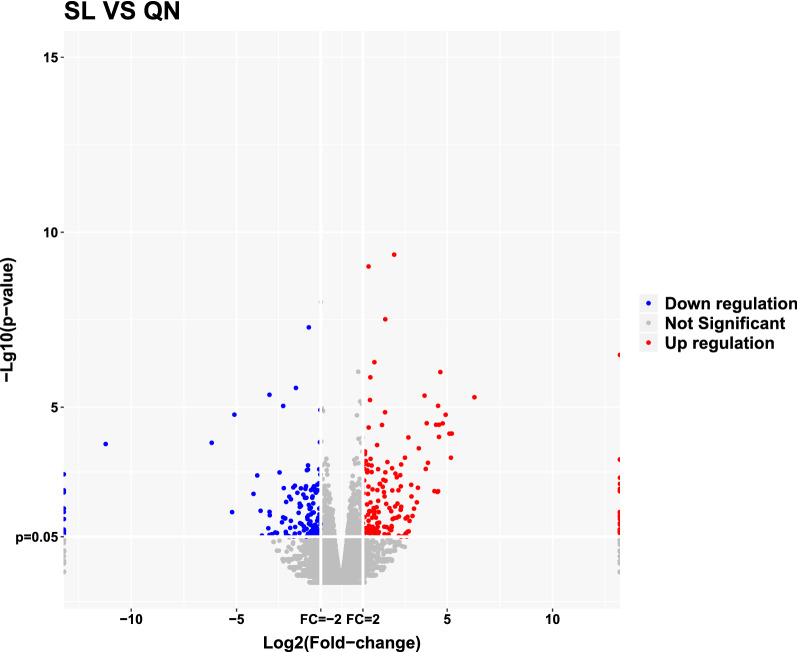
Volcano plot of differentially expressed mRNAs in the aging group and the 9-month control group. Normalized fold change and P values (aging group/9-month control group) were used to construct the volcano plots (n = 3). The y-axis and x-axis represent the *P* value and fold change, respectively. The red and blue dots represent significantly upregulated and downregulated mRNAs, respectively. The gray dots represent no statistically significantly altered mRNAs

A total of 326 mRNAs were significantly altered in the aging rat hippocampus compared to the 9-month-old control. Among these, 182 mRNAs were upregulated, while 144 mRNAs were downregulated. The most upregulated mRNA was AY172581.16, with an FC of 78.43 compared to the 9-month control. The most downregulated mRNA was Mt-nd4l, with an FC of 0.000421554 compared to the 9-month control. The top 20 upregulated and 20 downregulated mRNAs in the aging rats are listed in Table 2. The clustering analysis and volcano plot visualization showed dramatically different expression levels of mRNAs in the aging and 9-month control groups (Figs. 1 and 2, Additional file 1).

**Table 2:** Top 40 differentially expressed mRNAs determined by sequencing analysis

Gene name	P-value	Fold change	log2FC	Regulation	Location
AY172581.16	0.00000517	78.43307244	6.293390211	Up	MT:9800–9867
Sostdc1	0.0000565	37.20473726	5.217414425	Up	6:55812747–55817066
AABR07012061.2	0.000276717	36.19501976	5.177719299	Up	2:184544307–184570387
Ttr	0.0000568	34.47348865	5.107415399	Up	18:15532963–15540177
Tmem72	0.0000164	30.39692823	4.925853634	Up	4:148817514–148845267
Kl	0.0000289	27.60819556	4.787024693	Up	12:943006–987551
Mfrp	0.000000987	25.5258481	4.67388699	Up	8:48437918–48443421
Cldn2	0.0000317	24.44508965	4.611472791	Up	X:111122552–111137769
Slco1a2	0.0000702	24.40311831	4.608993607	Up	4:176445858–176528110
Cltrn	0.00250806	23.73775702	4.569111716	Up	X:32118054–32153794
Slc4a5	0.00000909	23.71043816	4.567450418	Up	4:114918488–115002300
LOC103690108	0.002629239	23.23285905	4.538094799	Up	20:3791407–3794027
F5	0.0000316	22.1951533	4.472172768	Up	13:82479998–82535534
Adipoq	0.00248908	21.02293971	4.393892515	Up	11:81330293–81344488
Steap1	0.000387301	16.99301725	4.086870133	Up	4:25435873–25446461
Col8a1	0.0000286	16.30922607	4.027616418	Up	11:44877859–45007891
AABR07049499.1	0.000579615	15.7977972	3.981651502	Up	5:124442293–124542156
Clic6	0.00000466	15.18118829	3.924212816	Up	11:32655616–32699382
Aqp1	0.000149122	12.57562744	3.652558478	Up	4:85551502–85569360
AABR07044362.6	0.001995124	12.21190348	3.610216186	Up	20:3134704–3135301
Mt-nd4l	0.000112407	0.000421554	− 11.211995	Down	MT:9870–10166
Igh-6	0.000103603	0.013803597	− 6.17881192	Down	6:138092131–138093643
Lhx8	0.009915014	0.027012853	− 5.21021016	Down	2:260574190–260596777
AABR07060872.1	1.62557E−05	0.029115265	− 5.10208043	Down	4:98337367–98523473
Cdcp1	0.002985718	0.054583323	− 4.19539596	Down	8:132260029–132296661
Gml	0.0008896	0.061732641	− 4.01782267	Down	7:116039715–116063098
AABR07034739.1	0.009103287	0.068974927	− 3.85778416	Down	11:86092468–86092779
AY172581.11	0.046440847	0.072127105	− 3.79331468	Down	MT:11665–11735
Ifna16l1	0.028680701	0.088881353	− 3.49197541	Down	5:107447061–107447636
AABR07051532.2	0.044424285	0.092347701	− 3.43678014	Down	3:16413080–16413632
AC111885.1	4.39529E−06	0.092360024	− 3.43658764	Down	14:78939961–78973883
LOC100359515	0.009751355	0.09260067	− 3.43283357	Down	10:64762907–64790306
Ctrb1	0.012207042	0.093264654	− 3.42252576	Down	19:43906292–43911057
LOC500354	0.042433549	0.100443212	− 3.31554803	Down	4:170820594–170821995
Krt85	0.037906846	0.111690915	− 3.16241625	Down	7:143161235–143167772
Olr392	0.039129216	0.118214938	− 3.08051575	Down	2:209581677–209582627
LOC100912028	0.000728748	0.128622522	− 2.95878481	Down	11:88569700–88570624
Slc12a1	0.019392253	0.139414033	− 2.84255231	Down	3:117421604–117498367
Pbk	0.013990766	0.144667143	− 2.78919081	Down	15:42489377–42500395
Cldn4	9.16883E−06	0.144974339	− 2.78613053	Down	12:24761210–24763005

### Expression profile of lncRNAs in the hippocampus of aging rats

**Fig. 3:**
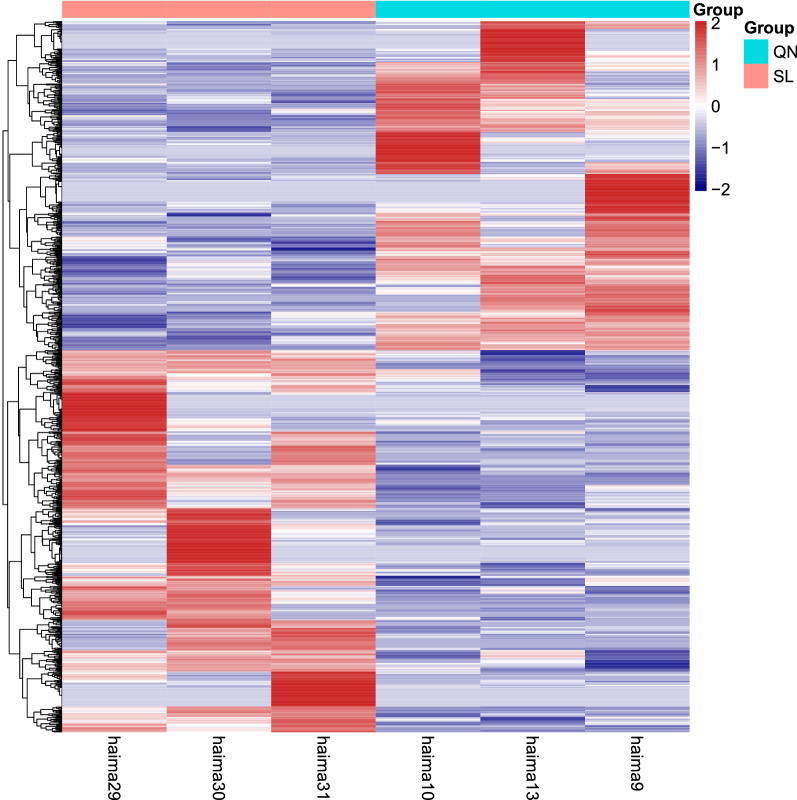
Heatmap of all differentially expressed lncRNAs in hippocampal tissues with aging. QN: 9-month control group; SL: aging group (n = 3). Each row represents one lncRNA, and each column represents one hippocampal sample. The relative lncRNA level is shown by the color scale. Red and blue represent high and low relative expression levels, respectively. The fold changes were normalized and scaled from − 2.0 to 2.0 by Z score

**Fig. 4:**
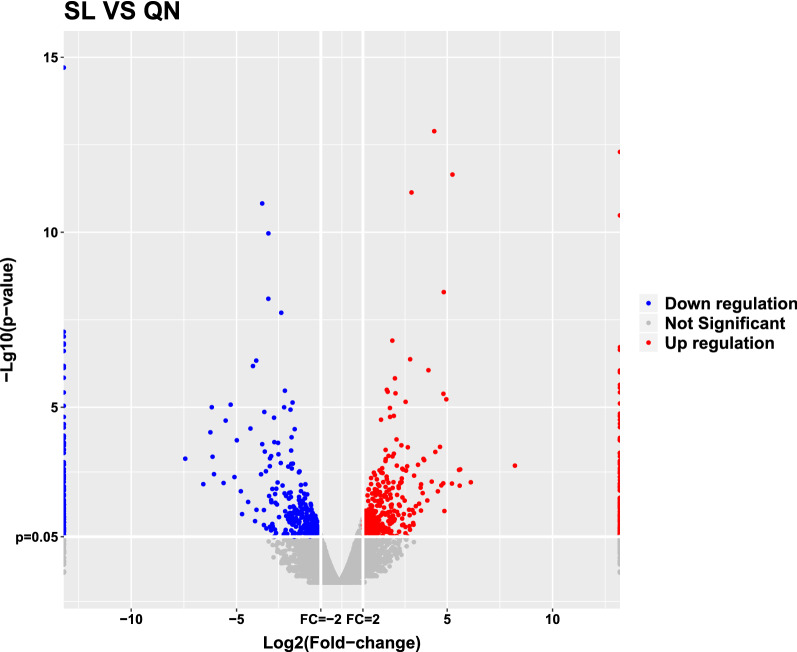
Volcano plot of differentially expressed lncRNAs in the aging group and the 9-month control group. Normalized fold change and *P* values (aging group/9-month control group) were used to constructvolcano plots (n = 3). The y-axis and x-axis represent the *P* value and fold change, respectively. The red and blue dots represent statistically significantly upregulated and downregulated lncRNAs, respectively. The gray dots represent nonsignificantly altered lncRNAs

With regard to lncRNAs, 1219 novel lncRNAs were identified and subsequently analyzed. These have not been reported in the past. A total of 832 lncRNAs were significantly altered in the aging rat hippocampus compared to the 9-month control. Among these, 447 lncRNAs were upregulated, while 385 lncRNAs were downregulated. The most upregulated lncRNA was MSTRG.28323.2, with an FC of 296.92 compared to the 9-month control. The most downregulated lncRNA was MSTRG.6082.3, with an FC of 0.0002 compared to the 9-month control. The top 20 upregulated and top 20 downregulated lncRNAs in the aging rats are listed in Table 3. The clustering analysis and volcano plot visualization showed dramatically different expression levels of lncRNAs in the aging and 9-month control groups (Figs. 3 and 4, Additional file 2).

**Table 3:** Top 40 differentially expressed lncRNAs determined by sequencing analysis

LncRNA_id	P-value	Fold change	Log2FC	Regulation	Location
MSTRG.28323.2	0.000466063	296.9235412	8.21394767	Up	X:29701682–29716271
MSTRG.3994.3	0.001390204	69.61342398	6.121293631	Up	10:20197654–20243996
NONRATT017026.2	0.000597039	49.08696512	5.617268067	Up	20:3132467–3133413
MSTRG.20241.2	0.001740925	48.25441553	5.592589057	Up	5:40229297–40231012
NONRATT013687.2	0.000616566	47.16378659	5.559607646	Up	18:15536213–15540087
MSTRG.11683.2	2.24104E−12	38.05815768	5.25013382	Up	17:58412803–58485208
NONRATT008064.2	0.001514701	37.35989038	5.223418317	Up	12:47433356–47438930
NONRATT028004.2	5.92765E−06	31.09144843	4.958445923	Up	8:115131458–115133401
MSTRG.2716.2	0.009227288	29.17942895	4.866879744	Up	1:214596038–214596434
NONRATT004028.2	5.12136E−09	28.5721171	4.836536034	Up	10:11720239–11721037
MSTRG.2246.19	4.1251E−06	28.26561591	4.820976229	Up	1:181259780–181295164
NONRATT021312.2	0.001490145	28.11568969	4.813303532	Up	4:60080944–60358416
MSTRG.12320.1	0.001706394	26.37822088	4.721275358	Up	18:31556917–31567379
ENSRNOT00000076888	0.000135499	25.2581862	4.658679137	Up	13:77784855–77822333
NONRATT003890.2	0.002523333	23.53802695	4.556921488	Up	1:266859985–266865673
NONRATT008903.2	0.000189188	21.66034671	4.436984431	Up	13:83220856–83227239
NONRATT006156.2	1.2934E−13	20.88437696	4.384352199	Up	10:94348384–94349845
NONRATT026294.2	0.001332002	19.23085369	4.265350903	Up	7:142238760–142243201
NONRATT028353.2	8.74005E−07	17.26288564	4.109601738	Up	8:23275331–23278375
NONRATT008900.2	0.004610768	16.6307113	4.055777969	Up	13:82535394–82536331
MSTRG.6082.3	0.000294801	0.00578063	-7.43455757	Down	11:63278719–63290256
NONRATT020530.2	0.001576394	0.010469053	− 6.57772522	Down	4:97635411–97760118
NONRATT028453.2	5.20E−05	0.013208114	− 6.24243173	Down	8:47255648–47256282
NONRATT002334.2	9.97E−06	0.013829031	− 6.1761561	Down	1:78844846–78851604
NONRATT003829.2	0.000259268	0.014193655	− 6.13861008	Down	1:264298811–264303479
NONRATT024688.2	0.00081681	0.014881369	− 6.07034894	Down	6:136358084–136363672
NONRATT005621.2	0.001459804	0.020390624	− 5.61595029	Down	10:57059768–57070891
NONRATT006931.2	2.40E−05	0.02184513	− 5.5165445	Down	11:43686192–43686776
NONRATT020371.2	8.43E− 06	0.025730471	− 5.28037834	Down	4:66275759–66284009
MSTRG.15789.2	0.000987355	0.029168593	− 5.09944037	Down	20:4780887–4924497
NONRATT025560.2	8.79E−05	0.031522719	− 4.98746419	Down	7:11999156–12000981
NONRATT006069.2	0.002514971	0.035986699	− 4.79639241	Down	10:89998285–90002189
MSTRG.1786.3	0.011304191	0.037498203	− 4.73703474	Down	1:140108223–140122003
NONRATT028403.2	0.00508097	0.045750127	− 4.45008045	Down	8:36754976–36771745
MSTRG.6345.3	4.06E−05	0.049424696	− 4.33862409	Down	11:83313317–83323596
NONRATT017309.2	6.64E−07	0.053638108	− 4.22059783	Down	20:13829727–13831971
NONRATT003291.2	0.017953853	0.057465961	− 4.12114853	Down	1:207997768–207998508
NONRATT017203.2	4.65E−07	0.059737031	− 4.06523065	Down	20:7417058–7427581
NONRATT015319.2	0.008518662	0.060236168	− 4.05322619	Down	2:150729332–150756141
NONRATT003889.2	0.000822712	0.06998689	− 3.83677149	Down	1:266859965–266866774

### Expression profile validation

**Fig. 5:**
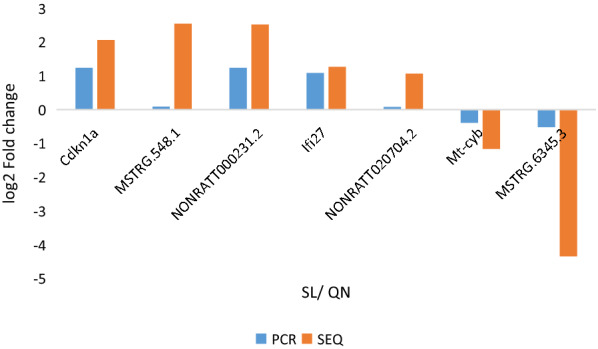
Validation of mRNA and lncRNA expression levels by RT‒PCR. QN: 9-month control group; SL: aging group (n = 3). mRNA and lncRNA expression levels were determined by RNA sequencing and RT‒PCR. Three parallel samples were assayed

To verify the validity of RNA sequencing, we randomly selected the differentially upregulated mRNAs of Cdkn1a and Ifi27, the downregulated mRNA of Mt-cyb, the differentially upregulated lncRNAs MSTRG.548.1, NONRATT000231.2 and NONRATT020704.2 and the downregulated lncRNA MSTRG.6345.3 that were abundantly expressed and exhibited significant changes for detection by RT‒PCR. The RT‒PCR results showed that the change trends of the selected mRNA and lncRNA levels determined by RT‒PCR were consistent with those determined by RNA sequencing (Fig. 5).

### GO function and KEGG pathway enrichment analyses of DE mRNAs

**Fig. 6:**
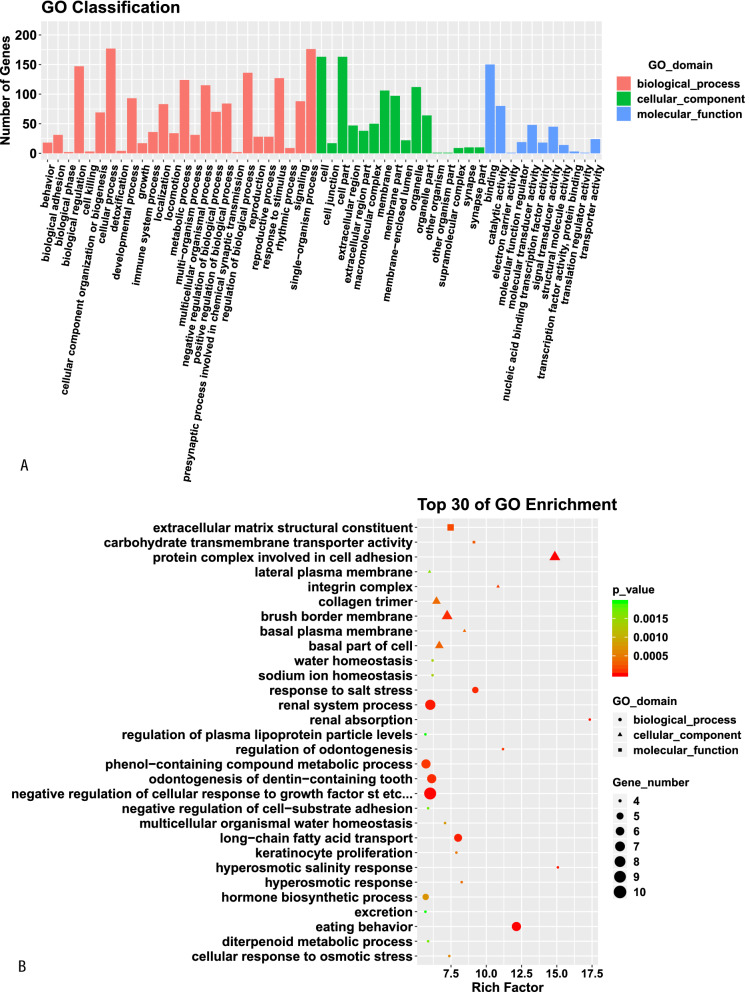

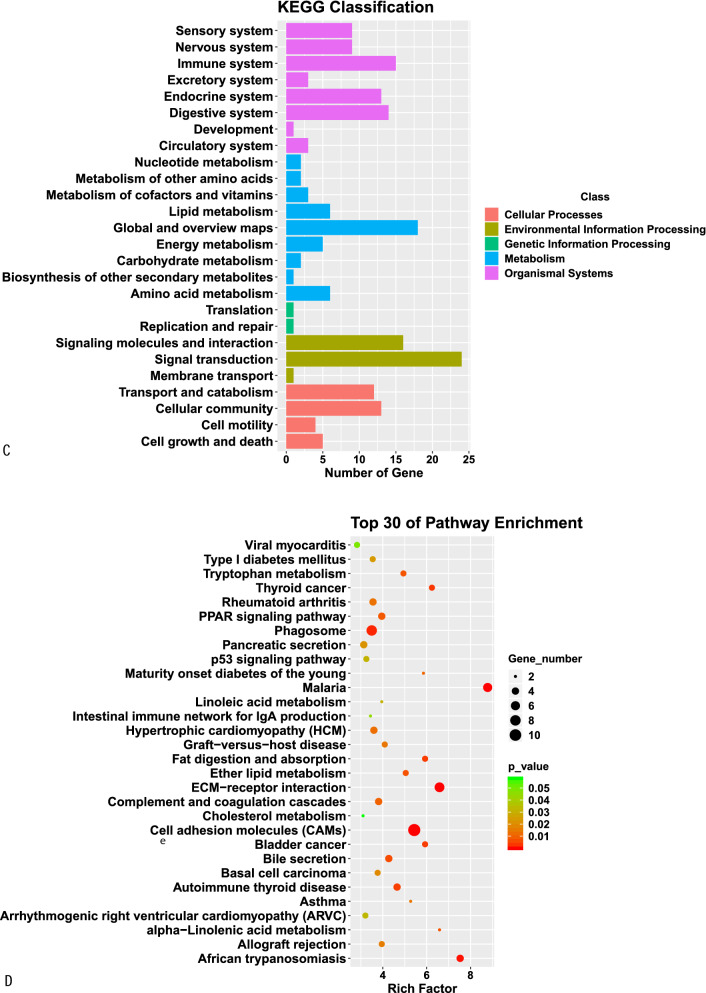
GO function and KEGG pathway classification (**A** and **C**) and enrichment (**B** and **D**) analyses of DE mRNAs. The enrichment value (− log10 (P value) was calculated and visualized to show the top 30 enriched GO terms (**B**), and top 30 enriched pathways (**D**)

GO analysis indicated that the most enriched mRNAs were related to negative regulation of cellular response to growth factor stimulus and long − chain fatty acid transport in the biological process category, collagen trimer, brush border membrane and protein complex involved in cell adhesion in the cellular component category, and extracellular matrix structural constituent in the molecular function category. KEGG pathway analysis showed that the top 30 differentially enriched KEGG pathways related to dysregulated mRNAs were phagosome, PPAR signaling pathway, p53 signaling pathway, ECM—receptor interaction, cell adhesion molecules, tryptophan metabolism and cell adhesion molecules (CAMs) (Fig. 6).

### GO function and KEGG pathway enrichment analyses of DE lncRNAs

**Fig. 7:**
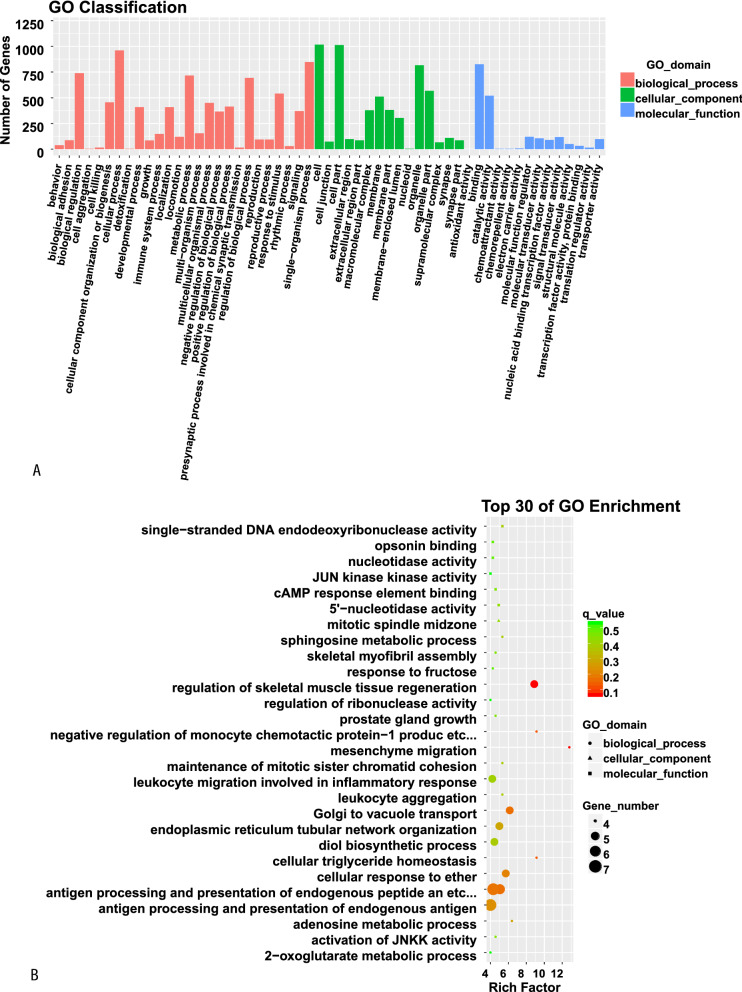

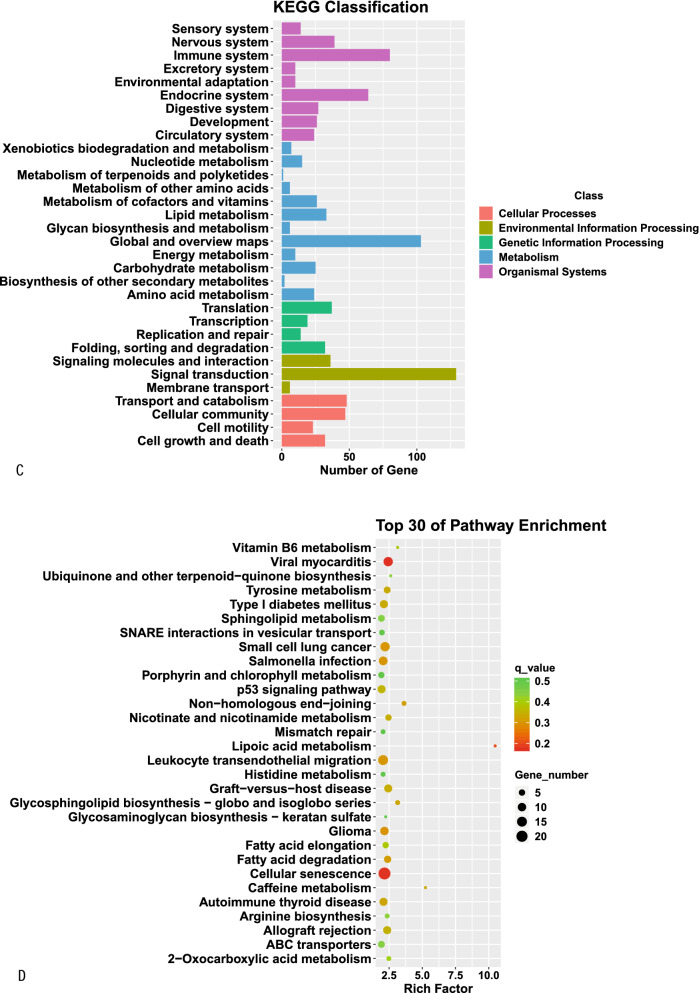
GO function and KEGG pathway classification (**A** and **C**) and enrichment (**B** and **D**) analyses of DE lncRNAs. The enrichment value (− log10 (P value) was calculated and visualized to show the top 30 enriched GO terms (**B**), and top 30 enriched pathways (**D**)

The target genes of lncRNAs were subjected to GO and KEGG analyses. GO analysis indicated that the most enriched target genes of lncRNAs were related to antigen processing and presentation of endogenous antigen, antigen processing and presentation of endogenous peptide antigen via MHC class Ib, regulation of skeletal muscle tissue regeneration and leukocyte migration involved in inflammatory response. KEGG pathway analysis showed that the top 30 differentially enriched KEGG pathways related to dysregulated lncRNAs were involved in cellular senescence, leukocyte transendothelial migration, the p53 signaling pathway and tyrosine metabolism (Fig. 7).

### Construction of the lncRNA‒mRNA coexpression network

**Fig. 8:**
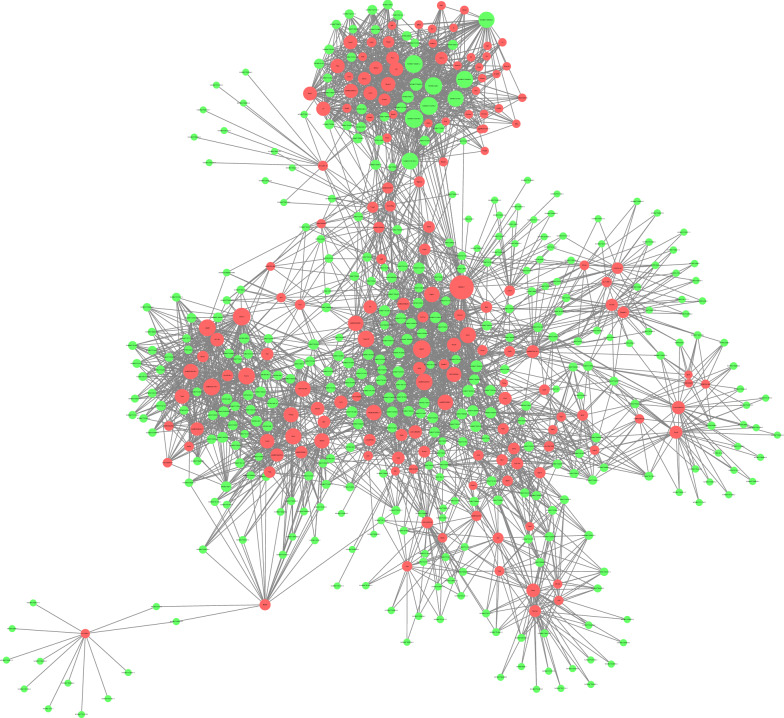
Coexpression network analysis of differentially expressed lncRNAs–mRNAs. After Pearson correlation analysis between DE lncRNAs and mRNAs, 516 mRNAs and their corresponding lncRNAs with a COR > 0.9 and *P* value < 0.01 were selected to construct a coexpression network of DE lncRNAs and mRNAs. Green dots represent lncRNAs, red dots represent mRNAs, the size of the circle represents the number of dots associated with them, and the more connections there are, the larger the dots

In total, 516 DE lncRNAs and their corresponding DE mRNAs were identified (*P* value < 0.01 and COR > 0.9), and the lncRNA‒mRNA coexpression network was constructed by Cytoscape 3.6.0 (Fig. 8). Among these lncRNAs, 52 lncRNAs may be involved in the regulation of the expression of Cdkn1a (24.445-fold change), which encodes a potent cyclin-dependent kinase inhibitor. MSTRG.548.1 (lncRNA, 5.840-fold change) and NONRATT000231.2 (lncRNA, 5.728-fold change) may be closely involved in the regulation of Cdkn1a expression.

### Construction of the lncRNA‒miRNA‒mRNA regulatory network

**Fig. 9:**
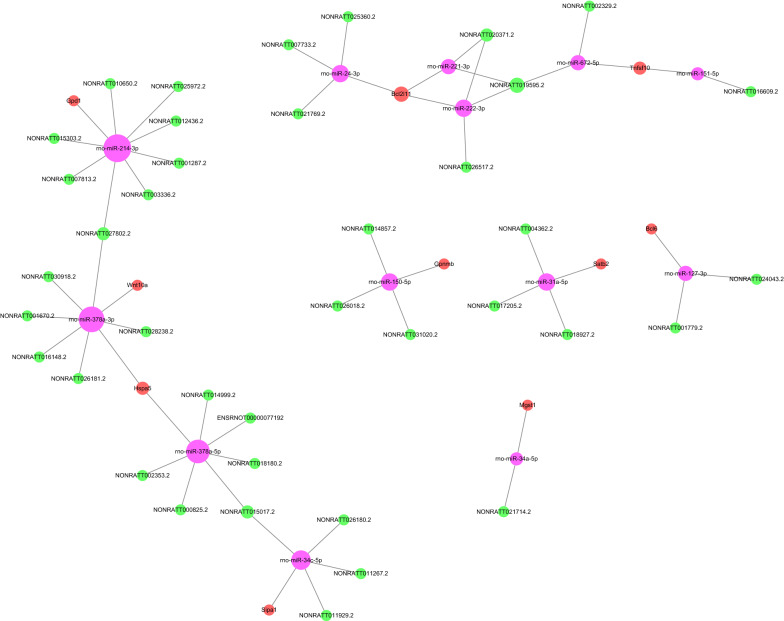
lncRNA‒miRNA‒mRNA ceRNA regulatory network. A ceRNA regulatory network of lncRNAs‒miRNAs‒mRNAs was constructed with DE lncRNAs and their predicted binding miRNAs and DE mRNAs and their predicted binding miRNAs by Cytoscape_3.6.0. Green dots represent lncRNAs, red dots represent mRNAs, pink dots represent miRNAs, and the size of the circle represents the number of dots associated with them; the more connections there are, the larger the dots

Because they competitively bind miRNAs as a miRNA sponge, lncRNAs could form a ceRNA network of lncRNAs‒miRNAs‒mRNAs to boost the expression of miRNA target genes. Based on the regulatory miRNA‒mRNA and lncRNA‒miRNA pairs, a lncRNA‒miRNA‒mRNA network was constructed. A total of 58 lncRNA‒miRNA–mRNA target pairs were identified, including 38 lncRNAs, 13 miRNAs, and 10 mRNAs (Fig. 9).

## Discussion

In this investigation, high-throughput sequencing revealed DE mRNAs and lncRNAs in the rat hippocampus during the aging process. In comparison to the 9-month control group, 326 mRNAs and 832 lncRNAs in the hippocampus of naturally aging rats showed significantly changed levels. Among them, 447 lncRNAs and 182 mRNAs were upregulated, and 385 lncRNAs and 144 mRNAs were downregulated. The results showed that the RT‒PCR data were almost completely consistent with the sequencing outcomes, which confirmed sequencing outcome reliability. In the process of aging, the synthesis of protein molecules related to cognitive function is reduced. Downregulation of lncRNAs may reduce the ability to protect mRNAs from degradation and reduce mRNA levels by reducing the inhibitory effect of lncRNAs on miRNAs. Upregulation of lncRNAs may upregulate the expression of protein molecules in a compensatory manner during natural aging. These DE lncRNAs and mRNAs may hold the key to the alterations in cognitive function, metabolism, function and hippocampal structure resulting from natural aging.

To better understand the potential mechanisms and biological roles of DE mRNAs in aging rats, we conducted GO and KEGG pathway enrichment analyses. GO analyses indicated that the most enriched mRNAs were associated with negative modulation of cellular response to GFS and long-chain fatty acid transport in the biological process category, collagen trimer, basal part of cell and basal plasma membrane in the cellular component category, and extracellular matrix structural constituent in the molecular function category. KEGG pathway analyses of the DE genes also revealed some key pathways associated with phagosomes, cell adhesion molecules, ECM—receptor interactions, p53 SP, PPAR SP, tryptophan metabolism and cell adhesion molecules (CAMs). The p53 SP and PPAR SP have previously been identified to be related to aging [25, 26]. Investigations in multiple organisms have demonstrated that tryptophan metabolism is a powerful regulator of age-associated disorders and lifespan [27]. The phagosome [28] and ECM-receptor interaction [29] are the most typical pathways associated with aging. By recruiting neurotransmitter receptors, scaffolding proteins and synaptic vesicles, closed with synaptic plasticity, CAMs have been confirmed to increase synaptic strength [30].

Among noncoding transcripts, lncRNAs have recently emerged as important regulators of the molecular pathways underlying age-related phenotypes. Interestingly, DE lncRNAs have been implicated in healthy aging [31, 32] and neurodegenerative and developmental disorders [33], raising the question of whether lncRNAs play a role in human brain aging. In the investigation, GO analyses indicated that the most enriched lncRNA target genes were related to antigen processing and presentation (APP) of endogenous peptide antigens and of endogenous antigens through MHC class Ib, modulation of skeletal muscle tissue regeneration and leukocyte migration associated with the inflammatory response. KEGG pathway analyses revealed that the top 30 differentially enriched pathways associated with dysregulated lncRNAs were involved in cellular senescence, leukocyte transendothelial migration, the p53 signaling pathway and tyrosine metabolism.

The mechanism of action of lncRNAs includes cis/trans gene regulation, nuclear structure organization and protein and RNA interaction and regulation [34]. In these two regulatory approaches, mRNAs and lncRNAs are coexpressed in neurons. By predicting the coexpression of mRNAs and lncRNAs, we can predict the possibility of some regulation or interaction between them. In the investigation, 561 lncRNAs related to DE mRNAs were predicted. This suggests that the lncRNAs are involved in DE mRNA regulation, but this would need to be verified through further experiments. Among these lncRNAs, 52 lncRNAs may be involved in the regulation of the expression of Cdkn1a (24.445-fold change), a major hallmark of senescence in most cells, which encodes a potent cyclin-dependent kinase inhibitor. Analysis of the data revealed MSTRG.548.1 and NONRATT000231.2 may be closely involved in the regulation of Cdkn1a expression. Therefore, further research is necessary to better understand the regulation of these networks.

Acting as molecular sponges for miRNAs via shared miRNA response elements (MREs), ceRNAs are groups of noncoding RNAs, mRNAs and other RNAs competing with miRNAs at the posttranscriptional level, thus modulating downstream molecular pathways and regulating mRNA expression. The ceRNA network links the role of protein-encoding mRNAs to the role of noncoding RNAs. In a target transcript, miRNAs binding to MREs decrease miRNA suppressor activity against other target genes. In theory, all transcripts containing one or more MREs can play a role as ceRNAs. Hence, the ceRNA modulation theory predicts a widespread posttranscriptional modulation pattern of gene expression. An in-depth investigation of the ceRNA regulatory mechanisms will help elucidate the disorder-related pathogenesis.

The lncRNA FLJ46906 binds to the transcription factors AP-1 and NF-κB and modulates the expression of aging-related genes [35]. Meanwhile, the lncRNA NEAT1 is involved in neurodegeneration, and suppression of this lncRNA in the hippocampus enhances memory in elderly mice by repressing neuronal histone methylation [36]. Previous investigations have shown that lncRNAs modulate target gene expression through the ceRNA network and are involved in the development of age-associated disorders [37, 38]. lncRNA-ES3 suppresses miR-34c-5p expression through direct interaction, and knockdown of this lncRNA inhibits the senescence of vascular smooth muscle cells, regulated through the lncRNA-ES3/miR-34c-5p/Bcl-2 modifying factor axis [39]. The lncRNA NONMMUT 055,714 constitutes a miRNA-7684-5p sponge that protects against postoperative cognitive dysfunction [40].

In this study, 58 lncRNA–miRNA–mRNA target pairs were confirmed, comprising 10 mRNAs, 13 miRNAs and 38 lncRNAs in rat hippocampal tissue. miR-214-3p, miR-378a-3p, miR-34c-5p, miR-24-3p, miR-222-3p, miR-150-5p, miR-31a-5p, miR-221-3p, miR-127-3p, miR-672-5p, and miR-34a-5p were identified to be related to aging processes in previous investigations. After comparing all total RNAs acquired from 13 old and 15 young individuals for validating the FC by employing quantitative RT-PCR, miR-24-3p was confirmed as a novel candidate aging biomarker [41]. By applying miR-378a-3p to study ‘aging miRNA’ profiles, patients were classified into two distinct groups presenting obviously different outcomes for some clinical/biological aging parameters [42]. In neurons of the cortex and hippocampus, miR-150-5p has previously been identified as deregulated in brain tissues in AD models [43]. Functional luciferase assays implied that mir-31a-5p in the hippocampus can modulate the expression of the interleukin 1 receptor antagonist and Mt1a [44]. miR-34a-5p was confirmed as an exosomal transfer RNA for inducing cardiac senescence-related injury, and suppressing miR-34a-5p in macrophages decreased the exosome PD-1 suppressor-induced prosenescent impact in cardiomyocytes [45]. Circulating miR-127-3p is a potential biomarker for differential diagnoses in frontotemporal dementia [46]. The results revealed that miR-31a-5p acts as an important regulator in the age-associated bone marrow microenvironment through the influence of osteoblastic and osteoclastic differentiation and that it can be a potential therapeutic target for age-associated osteoporosis [47].

Therefore, we speculated that the DE lncRNAs associated with aging in this study could hold the key to hippocampal senescence through the ceRNA network. Our research is only the beginning, and there remain many challenges to be addressed in the future. The mechanism by which DE lncRNAs regulate brain aging through the ceRNA network will be further verified. We will validate the ceRNA network and then search for meaningful pathways and biological processes based on the GO and KEGG results, identify the target mRNAs, and study the lncRNAs and miRNAs regulating the target mRNAs using gene knockdown or siRNA techniques in future work.

## Conclusions

In this study, we found specific lncRNAs and mRNAs in the hippocampus of natural aging model rats, as well as abnormal regulatory ceRNA networks. However, our current research has some limitations. A small specimen size can cause improper prediction of DE mRNAs and DE lncRNAs. In future research, a larger specimen size to verify our current outcomes is needed. Further experimental studies are also needed to compare the DE genes differences between male and female rats. In this study, 58 lncRNA‒miRNA‒mRNA target pairs were confirmed in the hippocampus, which may be involved in brain aging. Nonetheless, experiments are required to verify how lncRNAs modulate mRNAs via miRNAs. To date, this is the first high-throughput sequencing analysis of the expression profiles of lncRNAs and mRNAs in natural aging rats. Our results are helpful for understanding possible mechanisms of natural brain aging and offering a promising target to address aging.

## Supplementary Information

**Additional file 1:** The dramatically differentially expressed mRNAs in the aging and 9-month control groups.

**Additional file 2:** The dramatically differentially expressed lncRNAs in the aging and 9-month control groups.

## Data Availability

The datasets generated during the current study are available in the GSA repository (CRA007007, https://ngdc.cncb.ac.cn/gsa/s/p4q61kSb).

## References

[1] Xia X, Chen W, McDermott J, Han JJ (2017). Molecular and phenotypic biomarkers of aging. F1000Res.

[2] Lara J, Sherratt MJ, Rees M (2016). Aging and anti-aging. Maturitas.

[3] Capilla-Gonzalez V, Herranz-Pérez V, García-Verdugo JM (2015). The aged brain: genesis and fate of residual progenitor cells in the subventricular zone. Front Cell Neurosci.

[4] Kour S, Rath PC (2016). Long noncoding RNAs in aging and age-related diseases. Ageing Res Rev.

[5] Grammatikakis I, Panda AC, Abdelmohsen K, Gorospe M (2014). Long noncoding RNAs(lncRNAs) and the molecular hallmarks of aging. Aging (Albany NY).

[6] Knauss JL, Sun T (2013). Regulatory mechanisms of long noncoding RNAs in vertebrate central nervous system development and function. Neuroscience.

[7] Barry G (2014). Integrating the roles of long and small non-coding RNA in brain function and disease. Mol Psychiatry.

[8] Briggs JA, Wolvetang EJ, Mattick JS, Rinn JL, Barry G (2015). Mechanisms of long non-coding RNAs in mammalian nervous system development, plasticity, disease, and evolution. Neuron.

[9] Liu S, Wang Z, Chen D, Zhang B, Tian RR, Wu J, Zhang Y, Xu K, Yang LM, Cheng C, Ma J, Lv L, Zheng YT, Hu X, Zhang Y, Wang X, Li J (2017). Annotation and cluster analysis of spatiotemporal- and sex-related lncRNA expression in rhesus macaque brain. Genome Res.

[10] Kim SS, Lee SV (2019). Non-coding RNAs in *Caenorhabditis*
*elegans* Aging. Mol Cells.

[11] Liu P, Zhang X, Fu Q, Liu C, Luo Q, Yu P, Chen S, Zhang H, Qin T (2022). LINC01419 promotes the proliferation of hepatoma cells by recruiting XRCC5 and regulating its phosphorylation to repair DNA damage. Dis Markers.

[12] Huang Z, Wang H, Yang M (2022). Long non-coding RNA tumor protein 73 antisense RNA 1 influences an interaction between lysine demethylase 5A and promoter of tumor protein 73 to enhance the malignancy of colorectal cancer. Hum Cell.

[13] Tan P, Guo YH, Zhan JK, Long LM, Xu ML, Ye L, Ma XY, Cui XJ, Wang HQ (2019). LncRNA-ANRIL inhibits cell senescence of vascular smooth muscle cells by regulating miR-181a/Sirt1. Biochem Cell Biol.

[14] Wang Y, Liu Y, Jin Z, Liu C, Yu X, Chen K, Meng D, Liu A, Fang B (2022). Association between mitochondrial function and rehabilitation of Parkinson’s disease: revealed by exosomal mRNA and lncRNA expression profiles. Front Aging Neurosci.

[15] Faghihi MA, Zhang M, Huang J, Modarresi F, Van der Brug MP, Nalls MA, Cookson MR, St-Laurent G, Wahlestedt C (2010). Evidence for natural antisense transcript-mediated inhibition of microRNA function. Genome Biol.

[16] Shi XL, Zhao C, Yang S, Hu XY, Liu SM (2015). Moxibustion reduces ovarian granulosa cell apoptosis associated with perimenopause in a natural aging rat model. Evid Based Complement Alternat Med.

[17] Wenbo L (2018). Study on the effect of mild moxibustion at Shenshu point on DNA methylation modification of p16 gene in hippocampus of natural aging rats.

[18] Shafiei B, Shabani M, Afgar A, Rajizadeh MA, Nazari-Robati M (2022). Trehalose attenuates learning and memory impairments in aged rats via overexpression of miR-181c. Neurochem Res.

[19] Lomidze N, Zhvania MG, Tizabi Y, Japaridze N, Pochkhidze N, Rzayev F, Lordkipanidze T (2021). Aging affects cognition and hippocampal ultrastructure in male Wistar rats. Dev Neurobiol.

[20] van Praag H, Shubert T, Zhao C, Gage FH (2005). Exercise enhances learning and hippocampal neurogenesis in aged mice. J Neurosci.

[21] Yao ZH, Wang J, Shen BZ, Li YT, Yao XL, Zhang SF, Zhang Y, Hu JC, Xie YC (2020). Identification of a hippocampal lncRNA-regulating network in cognitive dysfunction caused by chronic cerebral hypoperfusion. Aging (Albany NY).

[22] Zhou C, Zhao W, Zhang S, Ma J, Sultan Y, Li X (2022). High-throughput transcriptome sequencing reveals the key stages of cardiovascular development in zebrafish embryos. BMC Genomics.

[23] Betel D, Wilson M, Gabow A, Marks DS, Sander C (2008). The microRNA.org resource: targets and expression. Nucleic Acids Res.

[24] Naghsh-Nilchi A, Ebrahimi Ghahnavieh L, Dehghanian F (2022). Construction of miRNA–lncRNA–mRNA co-expression network affecting EMT-mediated cisplatin resistance in ovarian cancer. J Cell Mol Med.

[25] Von Muhlinen N, Horikawa I, Alam F, Isogaya K, Lissa D, Vojtesek B, Lane DP, Harris CC (2018). p53 isoforms regulate premature aging in human cells. Oncogene.

[26] Cai Y, Liu H, Song E, Wang L, Xu J, He Y, Zhang D, Zhang L, Cheng KK, Jin L, Wu M, Liu S, Qi D, Zhang L, Lopaschuk GD, Wang S, Xu A, Xia Z (2021). Deficiency of telomere-associated repressor activator protein 1 precipitates cardiac aging in mice via p53/PPARα signaling. Theranostics.

[27] Wang D, Ye J, Shi R, Zhao B, Liu Z, Lin W, Liu X (2022). Dietary protein and amino acid restriction: roles in metabolic health and aging-related diseases. Free Radic Biol Med.

[28] Vieira OV, Botelho RJ, Grinstein S (2002). Phagosome maturation: aging gracefully. Biochem J.

[29] Wang H, Zhu X, Shen J, Zhao EF, He D, Shen H, Liu H, Zhou Y (2019). Quantitative iTRAQ-based proteomic analysis of differentially expressed proteins in aging in human and monkey. BMC Genomics.

[30] Thalhammer A, Cingolani LA (2014). Cell adhesion and homeostatic synaptic plasticity. Neuropharmacology.

[31] Barry G, Guennewig B, Fung S, Kaczorowski D, Weickert CS (2015). Long non-coding RNA expression during aging in the human subependymal zone. Front Neurol.

[32] Kour S, Rath PC (2017). Age-related expression of a repeat-rich intergenic long noncoding RNA in the rat brain. Mol Neurobiol.

[33] Wan P, Su W, Zhuo Y (2017). The role of long noncoding RNAs in neurodegenerative diseases. Mol Neurobiol.

[34] Tsagakis I, Douka K, Birds I, Aspden JL (2020). Long non-coding RNAs in development and disease: conservation to mechanisms. J Pathol.

[35] Yo K, Rünger TM (2018). The long non-coding RNA FLJ46906 binds to the transcription factors NF-κB and AP-1 and regulates expression of aging-associated genes. Aging (Albany NY).

[36] An H, Williams NG, Shelkovnikova TA (2018). NEAT1 and paraspeckles in neurodegenerative diseases: a missing lnc found?. Noncoding RNA Res.

[37] Liu ZQ, Zhang GT, Jiang L, Li CQ, Chen QT, Luo DQ (2021). Construction and comparison of ceRNA regulatory network for different age female breast cancer. Front Genet.

[38] Wei C, Luo T, Zou S, Zhou X, Shen W, Ji X, Li Q, Wu A (2017). Differentially expressed lncRNAs and miRNAs with associated ceRNA networks in aged mice with postoperative cognitive dysfunction. Oncotarget.

[39] Lin X, Zhan JK, Zhong JY, Wang YJ, Wang Y, Li S, He JY, Tan P, Chen YY, Liu XB, Cui XJ, Liu YS (2019). lncRNA-ES3/miR-34c-5p/BMF axis is involved in regulating high-glucose-induced calcification/senescence of VSMCs. Aging (Albany NY).

[40] Wei C, Sun Y, Wang J, Lin D, Cui V, Shi H, Wu A (2021). LncRNA NONMMUT055714 acts as the sponge of microRNA-7684-5p to protect against postoperative cognitive dysfunction. Aging (Albany NY).

[41] Machida T, Tomofuji T, Ekuni D, Maruyama T, Yoneda T, Kawabata Y, Mizuno H, Miyai H, Kunitomo M, Morita M (2015). MicroRNAs in salivary exosome as potential biomarkers of aging. Int J Mol Sci.

[42] Dalmasso B, Hatse S, Brouwers B, Laenen A, Berben L, Kenis C, Smeets A, Neven P, Schöffski P, Wildiers H (2018). Age-related microRNAs in older breast cancer patients: biomarker potential and evolution during adjuvant chemotherapy. BMC Cancer.

[43] Boese AS, Saba R, Campbell K, Majer A, Medina S, Burton L, Booth TF, Chong P, Westmacott G, Dutta SM, Saba JA, Booth SA (2016). MicroRNA abundance is altered in synaptoneurosomes during prion disease. Mol Cell Neurosci.

[44] Malan-Müller S, Fairbairn L, Hart S, Daniels WMU, Jalali Sefid Dashti M, Kidd M, Seedat S, Gamieldien J, Hemmings SMJ (2017). The role of microRNAs in the therapeutic action of D-cycloserine in a post-traumatic stress disorder animal model: an exploratory study. Psychiatr Genet.

[45] Xia W, Chen H, Chen D, Ye Y, Xie C, Hou M (2020). PD-1 inhibitor inducing exosomal miR-34a-5p expression mediates the cross talk between cardiomyocyte and macrophage in immune checkpoint inhibitor-related cardiac dysfunction. J Immunother Cancer.

[46] Piscopo P, Grasso M, Puopolo M, D'Acunto E, Talarico G, Crestini A, Gasparini M, Campopiano R, Gambardella S, Castellano AE, Bruno G, Denti MA, Confaloni A (2018). Circulating miR-127-3p as a potential biomarker for differential diagnosis in frontotemporal dementia. J Alzheimers Dis.

[47] Xu R, Shen X, Si Y, Fu Y, Zhu W, Xiao T, Fu Z, Zhang P, Cheng J, Jiang H (2018). MicroRNA-31a-5p from aging BMSCs links bone formation and resorption in the aged bone marrow microenvironment. Aging Cell.

